# Mass Spectrometric Identification of BSA Covalently Captured onto a Chip for Atomic Force Microscopy

**DOI:** 10.3390/ijms24108999

**Published:** 2023-05-19

**Authors:** Arina I. Gordeeva, Anastasia A. Valueva, Maria O. Ershova, Elizaveta E. Rybakova, Ivan D. Shumov, Andrey F. Kozlov, Vadim S. Ziborov, Maria G. Zavialova, Victor G. Zgoda, Yuri D. Ivanov, Alexander I. Archakov, Tatyana O. Pleshakova

**Affiliations:** Institute of Biomedical Chemistry (IBMC), 119121 Moscow, Russia; arina.atom@gmail.com (A.I.G.); varuevavarueva@gmail.com (A.A.V.); motya00121997@mail.ru (M.O.E.); zx2405@yandex.ru (E.E.R.); shum230988@mail.ru (I.D.S.); afkozlow@mail.ru (A.F.K.); ziborov.vs@yandex.ru (V.S.Z.); mariag.zavyalova@gmail.com (M.G.Z.); victor.zgoda@gmail.com (V.G.Z.); yurii.ivanov.nata@gmail.com (Y.D.I.); alexander.archakov@ibmc.msk.ru (A.I.A.)

**Keywords:** protein immobilization, atomic force microscopy, crosslinker, mass spectrometry, bovine serum albumin

## Abstract

Mass spectrometry (MS) is one of the main techniques for protein identification. Herein, MS has been employed for the identification of bovine serum albumin (BSA), which was covalently immobilized on the surface of a mica chip intended for investigation by atomic force microscopy (AFM). For the immobilization, two different types of crosslinkers have been used: 4-benzoylbenzoic acid N-succinimidyl ester (SuccBB) and dithiobis(succinimidyl propionate) (DSP). According to the data obtained by using an AFM-based molecular detector, the SuccBB crosslinker was more efficient in BSA immobilization than the DSP. The type of crosslinker used for protein capturing has been found to affect the results of MS identification. The results obtained herein can be applied in the development of novel systems intended for the highly sensitive analysis of proteins with molecular detectors.

## 1. Introduction

At present, mass spectrometry (MS) is the main technique for protein identification [1]. Protein identification is required in many biological and biomedical studies, including proteomic studies to inventory proteins in patient’s biological samples (proteomic profiling) and the search for potential biomarkers of pathologies. The identification of surface-anchored proteins is a separate area of proteomic analysis that is of great significance for biotechnology and bioengineering with regards to the investigation of the functional properties of surface-localized biosystems. Developed surfaces, such as chromatographic columns [2] and microbeads [3], as well as planar surfaces [4,5], in particular atomically smooth ones [6,7], can be considered in this respect.

Previously, we proposed the integration of MS with molecular detectors into a highly sensitive analytical system [8]. In the case of MS being combined with an atomic force microscopy (AFM)-based molecular detector, such an integration was called the AFM/MS approach [9]. This approach enables, on the one hand, the visualization and/or detection of protein complexes and, on the other hand, the identification of proteins in these complexes. The sensitivity of the analysis of several proteins using the AFM/MS approach was shown to be higher than that of the analysis of proteins in solution [10].

A key requirement for the application of AFM/MS is capturing the proteins to be studied onto an atomically smooth surface of AFM substrates. A protein can be captured onto the surface either by physical adsorption or by means of the formation of a covalent bond between chemically active surface groups and the protein’s functional groups. The second technique is preferable when the examined biomolecules are to be captured onto a small specific area of the sensor chip surface from a large volume of the analyzed solution. This approach, consisting of the irreversible capturing of the examined biomolecules from their solution onto the chemically activated, atomically smooth surface of the AFM substrate is called chemical fishing—analogous to the so-called molecular fishing [11,12]. In the fishing-based methods, the examined biomolecules are either chemically [13] or biospecifically [11,12,14] captured onto the sensor area of the surface of a specialized chip. In the case of chemical AFM-based fishing, the atomically smooth surface of the AFM substrate is treated with specialized agents, and the atomic smoothness of this surface remains unaffected during this treatment, which can include multiple steps. The substrate, whose surface is either partially or entirely activated with a chemical crosslinker, when ready for the capturing of the biomolecules to be examined, is called an AFM chip [8,13]—analogous to the sensor chip used in conventional molecular fishing [11,12]. The key difference between the chemical fishing and the conventional molecular fishing consists of the use of the chemical activation of the sensor area of the chip surface with a chemical crosslinker [13], instead of the use of antibodies [14]. In this way, the use of a crosslinker-activated chip, instead of an antibody-immobilized one, allows the analyst to overcome the thermodynamic limitation, which is imposed by the dissociation of antibody/antigen complexes on the chip surface on the lower limit of the antigen detection [14,15]. Chemical fishing is employed when it is necessary to non-specifically capture all the protein molecules from the analyzed solution. This is of use in the so-called “protein inventory systems” [16]. Protein inventory implies the detection of all the proteins present in the studied sample [16].

Previously, we demonstrated the good suitability of chemical fishing for highly sensitive protein analysis [13] and proposed developing this approach for protein inventory [10,13,17]. One of the promising areas is the use of nonspecific photocrosslinkers for the covalent binding of proteins [8], instead of the widely used crosslinkers with a terminal reactive succinimide group [18]. The possibility of the use of AFM/MS with photocrosslinkers was exemplified by the detection of human serum albumin (HSA) [8]. However, to implement the next step, i.e., to determine the sensitivity of this analysis, we needed to solve a methodological issue—whether the matrix-assisted laser derived ionization mass spectrometry (MALDI–MS) identification techniques developed for succinimide crosslinker-immobilized proteins can be used to analyze photoimmobilized proteins. In addition, MS analysis often requires multistep sample preparation; in the case of the highly sensitive AFM/MS approach, excessive steps may lead to sample loss or contamination. This requires the selection of the necessary, but sufficient, sample preparation steps, which do not interfere with the result, especially in the case of a highly sensitive analysis technique [19]. Zybailov discussed the features of the MS identification of covalently immobilized and crosslinked proteins [20].

The aim of the present study consists of the identification and analysis of differences in a peptide mixture composition with respect to the MS analysis of bovine serum albumin (BSA) covalently immobilized on the AFM chip surface via two different crosslinkers. We have studied how the type of chemical crosslinker, used for capturing the target protein onto amine-modified mica, affects the results of its MS identification. The results of the MS identification of BSA peptides have been compared for two different crosslinkers: a succinimide crosslinker, dithiobis(succinimidyl propionate) (DSP), and a photocrosslinker, 4-benzoylbenzoic acid N-succinimidyl ester (SuccBB). 

The rationale for choosing DSP and SuccBB crosslinkers was the following. DSP pertains to bifunctional succinimide esters, which are extensively used in biochemical studies [21]. Briefly, these compounds allow one to covalently bind examined protein biomolecules via their primary amine groups. The procedure of using these crosslinkers is relatively simple but has several disadvantages in the case of protein inventory systems. Namely, the number of primary amine groups on the surface of protein globules can be low, thus decreasing the probability of their capture by the succinimide groups of the chip surface. Furthermore, succinimide groups are subject to hydrolytic degradation [21], which has a considerable negative effect on the capturing efficiency during the long capturing time required at low protein concentrations [13]. DSP allows one to capture proteins from their solutions onto amine-modified mica even at low (down to sub-femtomolar) concentrations [13], while the surface roughness remains unaffected [13]. However, its capturing efficiency against albumins remains questionable [8], and further research in this direction should be performed. 

In protein inventory systems, the use of photoactivatable crosslinkers (photocrosslinkers) is preferable. Such crosslinkers include aryl azides, which are widely used in biochemical research [21]. Aryl azides were successfully used for obtaining modified antibodies, which are of use when biospecific irreversible capturing is required [14]. It should be emphasized that the use of additional irradiation sources is required in the case of photocrosslinkers, as opposed to DSP. The use of SuccBB is motivated by the fact that it readily binds proteins under relatively mild conditions at an activation wavelength of 350 to 370 nm [22], as opposed to aryl azides (which are activated at <300 nm wavelength [22]). Furthermore, the active groups formed by aryl azides and nitrenes degrade irreversibly if protein binding does not occur within 10^−13^ s [23]. In contrast, the activated benzophenone groups formed by SuccBB do not degrade irreversibly, while their lifetime can reach 120 µs [24]. The long lifetime of the active groups makes SuccBB attractive for its use in chemical AFM-based fishing in protein inventory systems, while the lifetime of the aryl azide active groups is very short. In addition, SuccBB binds proteins via their C-H groups [25], whose protein biomolecule numbers are very high, and this is favourable for their capture onto a surface activated with SuccBB. This is why SuccBB has been used in our experiments.

In our previous paper [8], we demonstrated that, at a 10^−9^ M concentration of albumin, only the SuccBB crosslinker exhibited sufficient capturing efficiency, allowing us to perform a reliable MS identification of the protein. Herein, we have studied these two crosslinkers at higher (10^−7^ M) albumin concentrations in order to find out whether the crosslinker type affects the results of the MS identification of the protein, captured onto an AFM substrate activated with this crosslinker (AFM chip).

In our experiments, we have employed BSA as a model protein since it is comprehensively characterized in the literature [26,27,28,29]. The molecular weight of BSA is 66.5 kDa [26], and its isoelectric point was reported to be between 4.2 and 5.5 depending on the salt concentration in the protein solution [27]. The amino acid sequence and the structure of this protein are well known [28,29], thus allowing us to perform a comprehensive analysis of the obtained results. This is why it is commonly used as a protein reference standard [30], and as a blocking agent in immunoassays [31,32]. BSA is the most abundant serum protein in bovine blood [33]. Accordingly, thanks to the cattle breeding industry, BSA is relatively cheap and readily available, and this is another reason for using this protein as a model in the experiments. 

The results of this study can be applied to the development of novel systems for highly sensitive AFM/MS analysis.

## 2. Results

### 2.1. AFM Imaging of the Surface with Covalently Immobilized BSA

**Figure 1:**
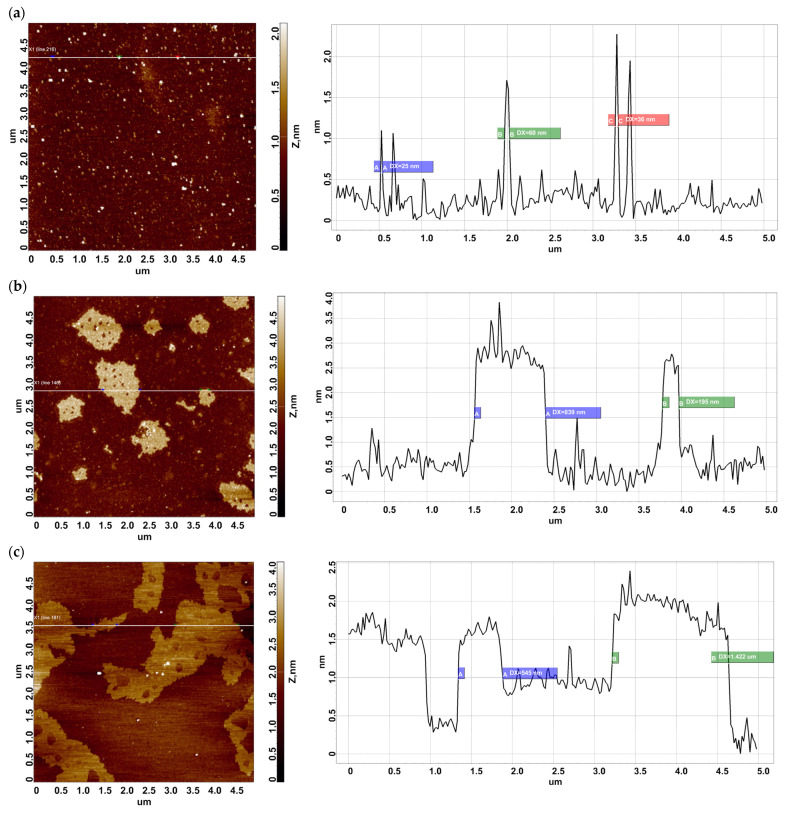
Typical AFM images of the mica surface with BSA immobilized via either DSP (**a**) or SuccBB (**b**,**c**) crosslinker. Left panels display AFM images of the chip surface. Right panels display cross-section profiles corresponding to the lines in the respective AFM images. The protein concentrations were 10^−7^ M (**a**,**b**) and 10^−9^ M (**c**).

Bovine serum albumin (BSA) was covalently immobilized on the AFM chip surface via either the succinimide crosslinker (DSP) or the photocrosslinker (SuccBB). Figure 1 displays the typical AFM images of the AFM chip surface with the immobilized protein.

As seen from Figure 1a, compact objects of ~1.5 nm in height are visualized on the AFM chip surface after the immobilization of BSA from a 10^−7^ M solution via the DSP crosslinker. When either 10^−7^ M (Figure 1b) or 10^−9^ M (Figure 1c) BSA solution and the SuccBB crosslinker are used for immobilization, layered structures of ~3 nm and ~2 nm in height, respectively, are observed on the AFM chip surface.

**Figure 2:**
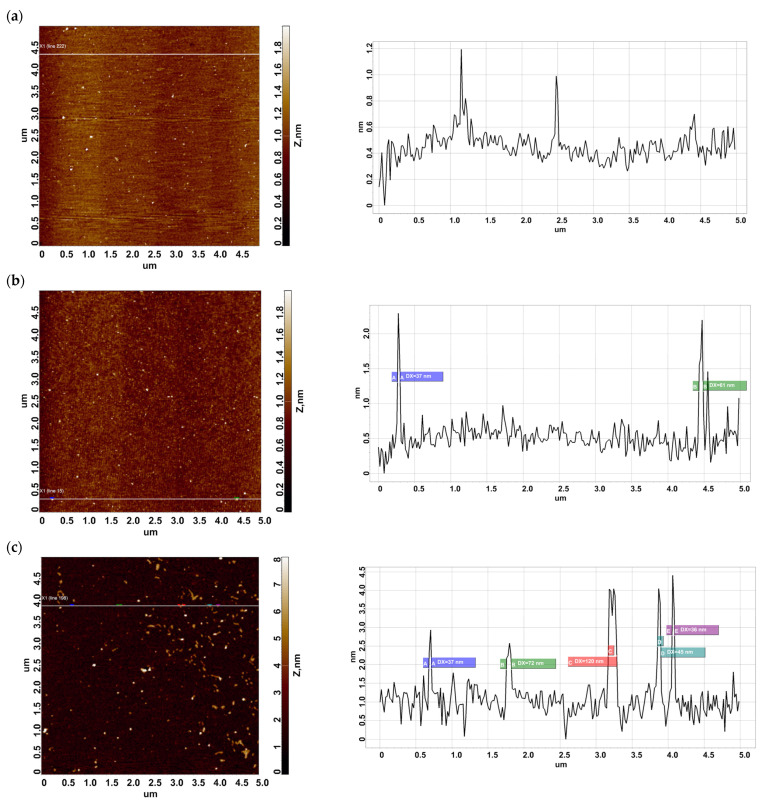
Typical AFM images of the mica surface obtained in control experiments. Left panels display AFM images of the chip surface. Right panels display cross-section profiles corresponding to the lines in the respective AFM images. (**a**) Control 1, crosslinker-activated AFM substrates are incubated in a protein-free solution (blank experiment). (**b**) Control 2, AFM substrate, whose surface was not activated with a crosslinker, was incubated in 10^−7^ M BSA solution. (**c**) Control 3, a SuccBB-activated AFM chip was incubated in 10^−7^ M BSA solution without UV irradiation.

Typical AFM images obtained in the control experiments are shown in Figure 2. 

The images shown in Figure 2 indicate that in Control 1 experiments (that is, in blank experiments after the incubation of a SuccBB-activated AFM chip in the protein-free buffer), an insignificant number of non-specific objects of >1 nm in height, a number lower than the noise level (500 objects/400 µm^2^ [13]), are observed on the surface. In Control 2 experiments (after incubation of the AFM chip, not activated with a crosslinker, in 10^−7^ M BSA solution under UV irradiation), a negligible number of objects of up to 2 nm in height are also visualized on the surface. In Control 3 (after incubation of the SuccBB-activated AFM chip in 10^−7^ M BSA solution without UV irradiation), laterally extended objects of up to 4 nm in height are visualized on the surface.

### 2.2. MS Identification of the Protein in Solution and in Eluates from the AFM Chip Surface

At this step of the study, two types of samples were analyzed. The first type was 10^−7^ M BSA solution. Eluates from the AFM chip surface, obtained after the capture of BSA from either 10^−7^ M or 10^−9^ M solution of this protein, pertained to the second type of samples. As mentioned above, two options were used in sample preparation for MALDI–MS analysis: y = 1 and y = 2, with and without the alkylation step, respectively. The results of the MALDI–MS analysis of both the BSA solution and the eluates from the AFM chip surface are summarized in Table 1 and Table S1 (please see Supplementary Information), which provides information on the number of peptides identified using the Peptide Mass Fingerprint method.

The analysis of 10^−7^ M BSA solution with the use of an alkylation step allowed us to identify 12 ± 3 peptides (Table 1, line 1). In parallel, 11 ± 1 peptides were identified when the alkylation step was excluded (Table 1, line 2).

The number of identified peptides in eluates from the AFM chip surface with DSP- and SuccBB-immobilized BSA for each set of experimental conditions is also shown in Table 1 (Lines 3–7).

As seen from Table 1 (Line 2, peptoset 2_1 and peptoset 2_2), when DSP was used to capture the protein, 3 ± 1 peptides were identified in eluates from the AFM chip surface with the use of alkylation (y = 1), and 5 ± 3 peptides were found in eluates from the chip surface in the absence of alkylation (y = 2).

When the SuccBB-activated chip was used to capture BSA from its 10^−7^ M solution, 9 ± 8 and 10 ± 6 peptides were identified in the eluates with and without the use of alkylation, respectively (Table 1, Line 3, peptoset 3_1 and peptoset 3_2). In the case of the 10^−9^ M BSA solution, the number of peptides decreased to 3 ± 2 in the absence of alkylation (Table 1, Line 4, peptoset 4_2). In order to additionally confirm the identification of the protein, MS/MS analysis of the BSA peptides was performed. The MS/MS spectra obtained in these experiments are presented in Supplementary Information (Figure S1).

### 2.3. MS Analysis of Samples Obtained in Control Experiments

The eluates of a tryptic peptide mixture from the surface of an AFM chip used in control experiments were also analyzed by MS. No peptides were identified in the eluates from the surface in Control 1. In Control 2, 3 ± 1 peptides were found in the absence of the alkylation step. In Control 3, 3 ± 1 peptides were identified.

## 3. Discussion

### 3.1. AFM Imaging Results

In this study, we have performed AFM imaging of BSA covalently immobilized on the surface of AFM chips via either a DSP or SuccBB crosslinker.

As one can see in Figure 1a–c and Figure 2b,c, objects of various shapes and heights have been observed on the surfaces in the working and control experiments. Namely, in the working experiment with the DSP-activated surface and 10^−7^ M BSA solution, the protein is immobilized mainly in the form of compact objects, whose heights are less than 2 nm. However, for BSA immobilized on the SuccBB-modified surface from either the 10^−7^ M or 10^−9^ M solution, extended fragments of a layer, whose heights can reach ~4 nm, have been observed. The visualized objects have been attributed to BSA molecules, since in the control experiments (Figure 2a,b), these objects are either absent or detected in much smaller amounts (Figure 2c). After scanning, all the AFM chips were subsequently subjected to MS analysis.

The results of AFM imaging demonstrate that, in the case of BSA captured onto a SuccBB-activated chip, a larger number of objects with a characteristic layered structure were observed on the surface in comparison with the case of the DSP-activated chip. This can be explained as follows.

The efficiency of protein capturing on solid substrates can be affected by a number of factors: different types of interactions between the protein and the surface [34], the pH and ionic strength of the protein solution [27,34], the protein aggregation state, the accessibility of the protein’s functional groups required for immobilization [21], the temperature [34], the lifetime of the functional groups of the chip surface in an excited state [23,24], and the degradation of these groups [21].

Norde [35] suggested that the understanding of protein adsorption on a surface requires “a novel approach which should zoom in on discrete events that occur at a sub-molecular level, taking into account nanoscopic heterogeneity of the surface and special details of the molecules”. The spatial distribution of a protein charge is highly asymmetric and can be characterized by the dipole moment of the protein. BSA has a dipole moment of 3.84 D, which is greater than that of water (1.84 D). These features can also affect the course of protein adsorption. Due to a non-uniform charge distribution over all BSA molecules [34], even for a negative net charge, there can be anomalous deposits on negatively charged surfaces.

Kuznetsova et al. studied the adsorption of BSA onto mica surfaces at various pH values [36]. At pH 3, BSA molecules were found to be predominantly adsorbed as a monolayer of 0.7 nm in height. At pH 6, there was multilayer adsorption, when subsequent layers of protein molecules adsorb onto their first monolayer, which is adsorbed directly onto the substrate surface. The authors explain this phenomenon by a long-range steric interaction, which exceeds the electrostatic repulsive forces between the protein and the surface.

Previously [8], using AFM, we failed to visualize human serum albumin (HSA) protein, which was immobilized onto an AFM chip surface from its 10^−9^ M solution in ultrapure water via a DSP crosslinker. Accordingly, we did not identify it using MS. For this reason, in the present study, we have increased the concentration of the protein solution by two orders of magnitude (up to 10^−7^ M) and have used a PBSD buffer (pH = 7.4) instead of deionized water to prepare the solution—that is, both the pH and the ionic strength of the protein solution have been changed. Increasing the protein solution concentration to 10^−7^ M has allowed us to successfully detect BSA molecules on a DSP-modified surface. However, the rate of hydrolysis of the DSP succinimide group was reported to be 300-fold higher than the rate of aminolysis [37,38]. For instance, upon the immobilization of IgG onto a DSP monolayer on gold substrate, the electrostatic, hydrogen, and Van der Waals interactions between the protein and the surface can prevail over their covalent interaction, even at a ~0.7 μM concentration of the protein [38]. 

The ionic strength of the buffer solution is another important factor, the influence of which on the interaction of a protein with a substrate can be very strong. Increasing the ionic strength leads to a stronger interaction between the protein molecules in buffer than in deionized water. This interaction in buffer significantly changes the thermodynamic properties of the protein solution, which can affect the interaction between the protein macromolecules and the substrate surface [39]. In the present study, the use of PBSD buffer for the preparation of the BSA solution is also expected to improve the efficiency of protein capturing with DSP, as compared with our previous results [8]. Indeed, at pH 7.4, the negative surface charge of BSA decreases with increasing ionic strength [27]. In [8], we justified that the surface of the DSP-activated AFM chip is charged negatively at near-physiological pH values. In this situation, increasing the ionic strength at pH 7.4 has allowed us to minimize the electrostatic repulsion between the AFM chip surface and the albumin macromolecules, thus favouring their approach to the chip surface with subsequent covalent capturing. 

Gaining a broader understanding of protein adsorption to improve the available materials is a challenge. Previously, it was shown that adsorbing BSA molecules form a multilayer film [40]. Notably, Close et al. [41] studied the adsorption of BSA on the surface of mica and determined the dependencies of BSA adsorption on pH. These authors observed a maximum adsorption of the protein onto the surface near the protein’s isoelectric point.

Herein, the different efficiencies of BSA capturing via DSP and SuccBB crosslinkers can be explained by another important factor—namely, by the availability of certain functional groups of the protein for interaction with the crosslinker-activated surface. DSP is specific for only primary amine groups [21], whose number in the protein molecule is relatively low. On the contrary, SuccBB interacts with C–H groups, whose number in the protein is much greater, thus leading to immobilization in the form of characteristic layered structures. This is why the efficiency of SuccBB in BSA capturing is much higher than in that of DSP, and this is what we have observed in our reported AFM experiments.

### 3.2. MS Analysis Results

The data obtained were analyzed in terms of the number of peptides identified by the Peptide Mass Fingerprint method and the composition of peptides identified for each of the examined samples.

In the MS analysis, the efficiency of the use of the alkylation and reduction step was estimated given the following inputs. Disulfide bonds connecting two cysteine residues are involved in the formation of the protein tertiary structure, which complicates the mass spectrometric analysis of peptides containing this amino acid residue. Proteolytic peptides containing preserved disulfide bonds have unexpected m/z values. This is why these peptides often remain unidentified [19]. To solve this problem, the sample preparation of protein solutions usually involves the reduction of cysteine residues, the cleavage of S–S bonds, and their subsequent modification, which blocks the re-formation of the disulfide bonds.

Obviously, different sample preparation methods are more or less suitable for each particular task (e.g., for a comparison of proteomes of biological samples, for studying posttranslational modifications of healthy and sick patients, or for mass spectrometric analysis from the surface) [36]. 

Herein, we have compared the results of the MS analysis of BSA samples performed either with or without the use of reduction and alkylation steps, designated as (y = 1) and (y = 2), respectively. We have expected that the disruption of disulfide bonds between two cysteine residues would allow the protein to unfold and expose free sites to trypsin, thus improving the efficiency of MS analysis. In the case of DSP, reducing agents would also cleave S–S bonds in the crosslinker and increase the efficiency of peptide desorption from the surface. We have used the number of identified peaks as a criterion for the efficiency and sensitivity of the analysis, at a signal-to-noise ratio (*S/N*) > 2.

The MS identification of BSA has been performed using the Peptide Mass Fingerprint method. The results have been visualized using RasMol 2.7.5.2 [42] software [43], which generates an animated 3D model using Protein Data Bank (PDB) data [44,45].

The data listed in Table 1 indicate that upon the use of DSP for the immobilization of the protein, the maximum number of identified peptides were four and eight peptides in samples with and without the alkylation step, respectively. With the use of SuccBB, the maximum number of peptides identified in eluates from the AFM chip surface was increased to seventeen and sixteen in samples analyzed with and without the alkylation step, respectively (Table 1, No. 3, peptoset 3_1 and peptoset 3_2). In the case of DSP, we expected that alkylation would increase the number of identified peptides due to the possible cleavage of the S–S bridge in the crosslinker for the reasons discussed above.

The low efficiency of the reduction and alkylation steps implemented on the AFM chip surface can be explained as follows. At neutral pH values, disulfide bonds in the BSA molecule are localized deep inside the protein globule, and are not exposed to the solvent, i.e., the protein is compactly folded in solution [46]. The only free cysteine residue (Cys34) is located in domain I, in the hydrophobic pocket of the molecule [47]. An additional step in sample preparation, which includes the reduction and alkylation of the protein in solution, is aimed at denaturing the native protein and reducing disulfide bonds, leading to an unfolding of the amino acid chain and the exposure of amino acid sites to cleavage by proteases.

The results of the MS identification were also expected to be affected by the interactions of the protein with the AFM chip surface [34]. The structure of the proteins is known to change upon their interaction with the surface [34,48]. Therefore, the alkylation and reduction step in this case did not significantly contribute to hydrolysis. For this reason, we excluded this step from the sample preparation for MS analysis in both the control experiments and the experiments with the 10^−9^ M BSA solution.

A comparative analysis of the results of the MS identification of BSA covalently immobilized on the AFM chip surface via the succinimide crosslinker (DSP) and the photocrosslinker (SuccBB) has revealed that the crosslinker type considerably affected the composition of the trypsinolytic mixture eluted from the surface. The data summarized in Table 1 indicate that the protein contents in eluates from the AFM chip surface were more than two-fold higher in the case of SucBB than DSP (Table 1, lines 2 and 3, respectively).

**Figure 3:**
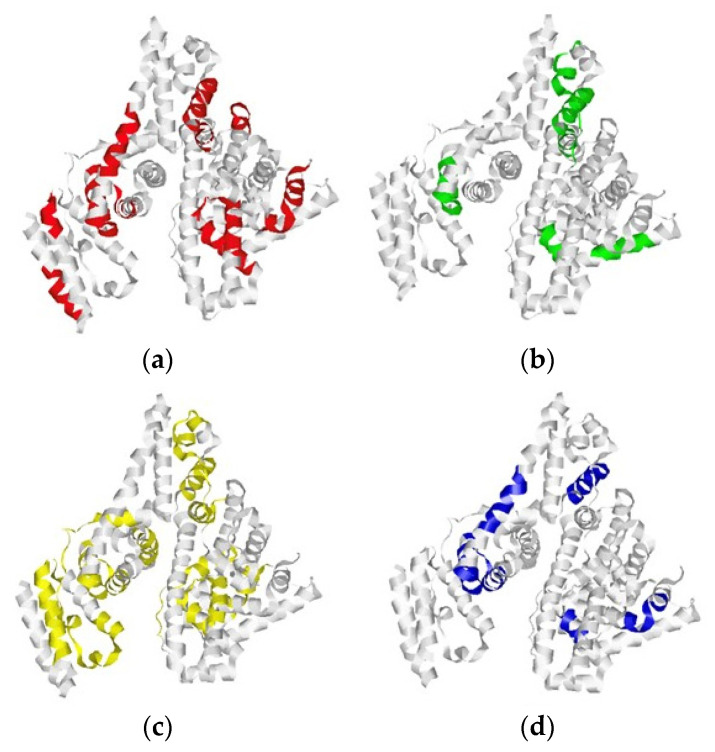
BSA structure represented using a RasMol script. BSA peptides identified by MS analysis are marked with colour. Samples were prepared using various crosslinkers, including alkylation of cysteine residues and without this step: (**a**) SuccBB crosslinker; (**b**) DSP crosslinker; (**c**) SuccBB crosslinker including alkylation of cysteine residues; (**d**) DSP crosslinker including alkylation of cysteine residues.

In order to understand the difference between the results of the MS identification of the protein immobilized via different crosslinkers, we have presented the BSA molecule whilst taking into account the data obtained from the MS analysis using the data from PDB [45] that was generated by RasMol [42,43] using a RasMol script. Figure 3 displays the protein structure, in which the peptides identified in the MS analysis are highlighted with colour.

As seen from Figure 3, the proteolytic mixture composition is different in cases when either DSP (Figure 3a) or SuccBB (Figure 3b) is used for the modification of the AFM chip surface to immobilize BSA from its 10^−7^ M solution. In the case of SuccBB, the percentage of detected peptides is higher, as indicated by the coloured fragments of the BSA macromolecule (Figure 3b). In this case, the best results are obtained with the use of the alkylation and reduction step, (Figure 3c) and (Figure 3d), since it allows one to detect more unique peptides.

We have suggested that SuccBB-immobilized BSA is oriented on the chip surface in such a way that more free sites for trypsin are available.

**Figure 4:**
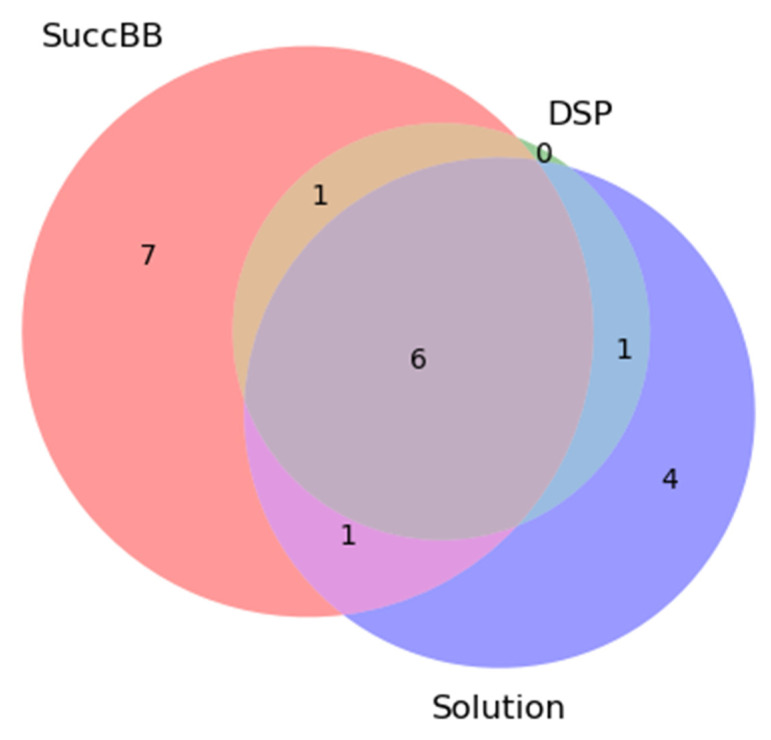
Results of MS identification of BSA are presented as a Venn diagram: peptides identified in analysis of 10^−7^ M solution (purple); in analysis of samples from the chip surface activated by DSP (green); and SucBB (pink).

In order to analyze the obtained data, we present below the difference in the peptoset compositions for DSP and SuccBB, and for the 10^−7^ M protein solution in the form of a Venn diagram, which is shown in Figure 4.

The BSA peptides identified were quantitatively compared using a three-circle Venn diagram (Figure 4). The dark lilac segment in the center of the diagram represents six common peptides identified in the solution and on the AFM chip surface with different crosslinkers. The peach segment represents seven peptides identified only in the case of SuccBB; the purple segment represents four peptides identified only in the solution; and the green segment represents unique peptides identified only in the case of DSP.

In Figure 4, one can see that, in the case of DSP and the 10^−7^ M protein concentration in the immobilization solution, all identified peptides have also been identified in other samples, in contrast to SuccBB.

## 4. Materials and Methods

### 4.1. Proteins

Bovine serum albumin (BSA) was from Sigma (Cat.#A6003; St. Louis, MO, USA). Porcine trypsin was from Promega Corp. (Cat.# V5111, Madison, WI, USA). Promega Sequencing Grade Modified Trypsin is a porcine trypsin modified by reductive methylation, rendering it resistant to proteolytic digestion.

A 10^−7^ M (0.1 µM) solution of BSA was prepared by dissolving the above-mentioned commercially available BSA in Dulbecco’s modified phosphate buffered saline (PBSD buffer). A concentration of 10^−9^ M was obtained by a hundredfold dilution of the 10^−7^ M solution with PBSD buffer.

### 4.2. Chemicals

The following reagents were used in the experiments: dithiobis(succinimidyl propionate) (DSP) (Pierce, Waltham, MA, USA), 4-benzoylbenzoic acid N-succinimidyl ester (SuccBB) (Sigma, St. Louis, MO, USA), 3-aminopropyltriethoxysilane (APTES) (Acros Organics, Fair Lawn, NJ, USA), dimethyl sulfoxide (DMSO) (Sigma, St. Louis, MO, USA), Emulgen 913 (Kao Atlas, Osaka, Japan), triethylammonium bicarbonate (TEAB) buffer (1 M, for HPLC; Sigma-Aldrich, St. Louis, MO, USA), 100% acetonitrile for HPLC (CAN) (Merck, Darmstadt, Germany), 2,5-dihydroxybenzoic acid (DHB) matrix (Bruker Daltonics, Bremen, Germany), 2-chloroacetamide (CAA; Sigma-Aldrich, Darmstadt, Germany), trifluoroacetic acid (TFA) and chloroacetamide (Sigma, St. Louis, MO, USA), and 0.5 M Tris(2-carboxyethyl) phosphine hydrochloride (Bond-Breaker TCEP solution, neutral pH) (Thermo Scientific, Waltham, MA, USA).

PBSD buffer was prepared by dissolving a salt mixture, commercially available from Pierce (Waltham, MA, USA), in ultrapure water. All solutions used in this study were prepared using deionized ultrapure water (resistivity, 18.2 MΩ × cm) obtained with a Simplicity UV system (Millipore, Molsheim, France).

### 4.3. Experiment Design

**Figure 5:**
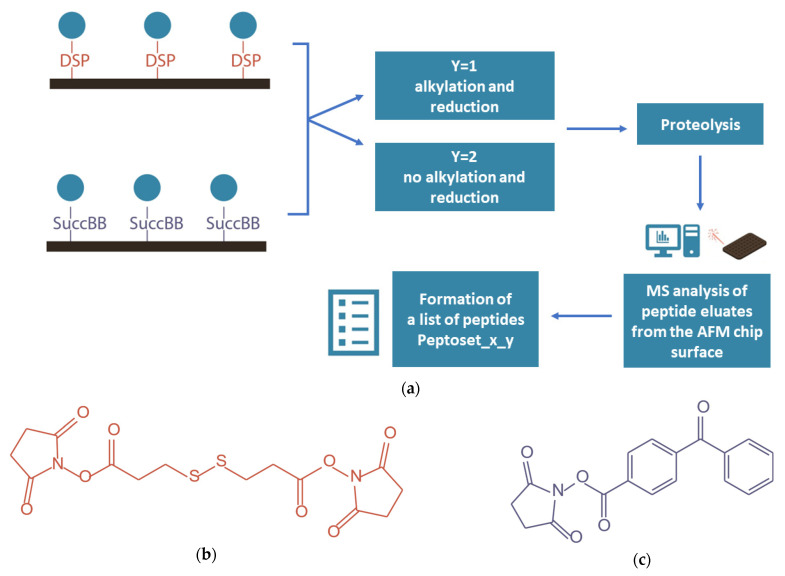
Workflow of MS analysis of BSA captured onto AFM chip surface (**a**), and structural formulas of crosslinkers DSP (**b**) and SuccBB (**c**).

The experiment design is shown in Figure 5.

BSA, covalently immobilized on a crosslinker-activated surface of mica AFM chips, underwent trypsinolysis on the chip surface. Then, a proteolytic mixture was eluted from this surface and applied onto a MALDI target for analysis of its peptide composition. 

Herein, a set of peptides identified in each sample has been called “peptoset_x_y”, where x is assigned a value of 1 to 4, depending on the analyte composition: 10^−7^ M BSA solution (x = 1); a proteolytic mixture from a DSP-modified AFM chip surface after capturing of BSA from its 10^−7^ M solution (x = 2); a proteolytic mixture from a SuccBB-modified AFM chip surface after capturing of BSA from its 10^−7^ M solution (x = 3); a proteolytic mixture from its SuccBB-modified AFM chip surface after capturing of BSA from a 10^−9^ M solution (x = 4). Y is assigned a value of 1 or 2 depending on the presence or absence of the sample preparation step for MS analysis: y = 1 if the alkylation and reduction step is involved; y = 2 if there is no alkylation and reduction step.

Briefly, the experiment comprised the following steps: protein immobilization via covalent binding to a crosslinker-activated surface, AFM imaging of the surface to confirm binding of the protein, sample preparation for MS analysis, and MALDI–MS for identification of peptide mixture composition. Sample preparation for proteomic MS analysis included protein reduction and alkylation of cysteine residues [19] followed by enzymatic hydrolysis [49]. Reduction of cysteine residues led to disruption of S–S-bonds and protein unfolding, which increased the number of free sites for hydrolysis [19].

The results of MS analysis were presented in the form of so-called “peptosets”, allowing us to identify both the surface-captured protein and the protein in solution. These results were analyzed using literature data on the structural properties of BSA in order to find the difference between proteolytic composition of this protein captured with the use of the two crosslinkers, DSP and SuccBB.

### 4.4. Modification and Activation of AFM Substrate Surface

Muscovite mica sheets (SPI, West Chester, PA, USA), cut into 7 × 15 mm pieces, were used as a material for AFM substrates. The surface of muscovite mica substrates was modified with (3-aminopropyl)triethoxysilane (APTES) using a vapour deposition technique developed by Yamada et al. [50].

To activate the substrate surface with the DSP crosslinker, 1.2 mM DSP solution in DMSO:EtOH (1:1) was mixed with PBSD buffer (10 mM, pH 7.4) at a 1:1 ratio (*v*/*v*). The activation solution volume was 1 mL. Next, the AFM substrate was immersed into an Eppendorf-type test tube containing 1 mL of the DSP solution, and incubated therein at room temperature and 600 rpm in a shaker for 10 min. After this incubation, the substrate was washed with 1 mL of 50% (*v*/*v*) ethanol for 10 min and dried in a nitrogen stream. The so-prepared AFM chips (that is, the AFM substrate with crosslinker-activated surface) were then immediately used for protein immobilization.

A 0.31 mM SuccBB solution was obtained by dissolving a weighed quantity of SuccBB in DMSO (final volume of the activation solution was 1 mL). Next, the AFM substrate was immersed into an Eppendorf-type test tube containing 1 mL of the SuccBB solution, incubated at room temperature, and placed at 120 rpm in a shaker for 18 h. After the incubation, the AFM substrate was washed once with 1 mL of DMSO:EtOH (1:1) at 40 °C for 30 min, and then twice with 1 mL of 50% (*v*/*v*) EtOH for 30 min. After the washing, the AFM chip was dried in a nitrogen stream and used for protein immobilization.

### 4.5. Covalent Capturing of the Protein

In the case of a DSP-activated surface, AFM chip was incubated in 1 mL of 10^−7^ M BSA solution in a rotating Eppendorf-type test tube at 25 °C and 600 rpm for 60 min.

In the case of a SuccBB-activated surface, AFM chip was incubated in 1 mL of either 10^−7^ M or 10^−9^ M BSA solution in a rotating test tube at 200 rpm under UV irradiation at a wavelength of ~365 nm for 1 h. The irradiation was performed with a UVP Crosslinker CL-3000L device (Analytik Jena US, Upland, CA, USA). The distance between the test tube and the UV light source was ~10 cm.

AFM chips, activated with either DSP or SuccBB and immobilized with the protein as described above, were then washed once with 1 mL of 0.01% aqueous solution of Emulgen 913 at 37 °C for 30 min, then twice with 1 mL of ultrapure water at 37 °C for 30 min, and dried in air.

Control experiments were performed as described above, but with the following modifications:

(1) In order to estimate the amount of non-protein particles adsorbed on the surface, blank experiments were performed; crosslinker-activated AFM chips were incubated in protein-free solution (Control 1);

(2) In order to estimate the amount of non-specifically (i.e., non-covalently) adsorbed protein macromolecules, AFM substrates, whose surface was not activated with a crosslinker, were incubated in 10^−7^ M BSA solution (Control 2);

(3) In order to estimate a change in adsorption properties of the substrate surface caused by crosslinker activation, the SuccBB-activated AFM chip was incubated in 10^−7^ M BSA without UV irradiation (Control 3).

In Control 2 and Control 3 experiments, solutions with the highest protein concentration (10^−7^ M BSA) were used.

### 4.6. AFM Scanning

In control and working experiments, the AFM chip surface was scanned in tapping mode using a Titanium atomic force microscope (NT-MDT, Zelenograd, Russia; the microscope pertains to equipment of “Human Proteome” Core Facility of the Institute of Biomedical Chemistry, supported by Ministry of Education and Science of Russian Federation, agreement 14.621.21.0017, unique project ID RFMEFI62117X0017). NSG10 cantilevers (“TipsNano”, Zelenograd, Russia), with a tip curvature radius of 6–10 nm, a resonant frequency of 47–150 kHz, and a force constant of 0.35–6.1 N/m, were used. The scan size was 5 × 5 µm (resolution, 256 × 256 points); at least 10 scans of different substrate areas were obtained for each substrate. A TGZ1 grating (Zelenograd, Russia) with a step height of 21.4  ±  1.5 nm was used for calibration of the microscope.

Processing of AFM images was performed using standard NovaPx 3.5.0 software and involved subtraction of the second-order plane. The height of AFM-detected objects was the main criterion of determination of their size [8,13,51,52].

### 4.7. Preparation of the AFM Chips for MS Measurements

In series 1 of working experiments on MS identification of BSA on the surface of AFM chips after the capturing from 10^−7^ M protein solution, alkylation and reduction followed by hydrolysis was used.

For alkylation and reduction of cysteine residues, 20 µL of TEAB, 2 µL of TCEP, and 4 µL of CAA were pipetted into a test tube with the AFM chip. The test tube was sealed with aluminium foil and incubated for 60 min at 80 °C and 1100 rpm.

The alkylation/reduction step was omitted in working experiments with 10^−9^ M BSA solution and in control experiments.

For trypsinolysis, each AFM chip was immersed into a solution containing 5 µL of trypsin (0.18 g/L), 20 µL of TEAB (50 mM), and 200 µL of deionized water so that the solution completely covered the chip. Trypsinolysis was performed in a shaker at 37 °C and 140 rpm for 18 h, and then in a heat chamber at 40 °C for 2 h, in a horizontal position so that the mica was covered with the buffer. Next, the chips immersed in a tryptic mixture were placed into a shaker, and incubated therein at 40 °C for 60 min. Then, the samples were shaken with a Sky Line vortex (Elmi Ltd., Rīga, Latvia) in MIX1 mode, and centrifuged at 9000 rpm for 10 min.

### 4.8. MS Measurements

MS analysis of protein peptides was performed using an AutoFlex III MALDI time-of-flight (TOF) mass spectrometer (Bruker Daltonics GmbH, Bremen, Germany) equipped with a nitrogen laser with an emission wavelength of 337 nm. The mass spectrometer was calibrated using a peptide calibration standard for the positive ion mode. The measured mass spectrum was between 660 and 4000 *m*/*z* at a pulse delay time of 200 ns. The peptide calibration standard included the following peptides: bradykinin (757.3992 Da), angiotensin II (1046.5420 Da), angiotensin I (1296.6853 Da), peptide P (1347.7361 Da), bombesin (1619.8230 Da), renin (1758.9326 Da), ACTH fragment 1–17 (2093.0868 Da), ACTH fragment 18–39 (2465.1990 Da), and somatostatin (3147.4714 Da).

For acquisition of sample mass spectra, a hydrolyzed mixture (2 μL) was spotted in layers with 1 μL of a DHB matrix (40 g/L, 50% ACN, and 50% TFA) onto an MTP AnchorChip 384 target plate in triplicate [53]. Mass spectra were processed using the FlexAnalysis 2.0 software (Bruker Daltonics GmbH, Bremen, Germany).

### 4.9. MS Data Processing

Acquired mass spectra were calibrated using the FlexControl 3.0 software (Bruker Daltonics GmbH, Bremen, Germany) (external calibration) and FlexAnalysis 2.0 (internal calibration); at least 2 trypsin autolysis peaks at 842.509 and 2211.104 were used for calibration in the linear mode (according to the manufacturer’s recommendations) and the quadratic mode with 3 or more points of internal calibration.

Further, a peptide map was generated in accordance with the BSA passport provided by the manufacturer. The UniProt service [54] was used to search for the desired protein P02769 (ALBU_BOVIN). To generate a peptide map, we used a web.expasy service [55] with two missing hydrolysis sites, potential modifications: Oxidation (M) [8,36].

For a peptide mixture produced by enzymatic cleavage of a protein, mass spectra are a specific “fingerprint” of the protein for its unambiguous identification. Experimental peptide masses are compared with database peptide masses calculated by applying enzymatic digestion rules to records in one of the major sequence databases, e.g., SwissProt [56].

The results obtained with the FlexAnalysis 2.0 software were compared with the peptide map using proteomics tools for mining sequence databases in conjunction with Mass Spectrometry experiments ProteinProspector v 6.4.5 [57]. The peptides identified by means of the BSA peptide map using the Peptide Mass Fingerprint method [58] were analyzed for the contents of human type I and type II keratins; the data for this protein were removed during processing of the spectra. The list of the identified BSA peaks included peptide masses with a monoisotopic mass accuracy of no more than 100 ppm.

A set of peptides obtained for each sample was designated as “peptoset_x_y”, where x was assigned a value of 1 to 4, depending on the analyte composition, namely: 10^−7^ M BSA solution (x = 1); a proteolytic mixture from the AFM chip surface, activated with the DSP crosslinker, after the immobilization of BSA from its 10^−7^ M solution (x = 2); a proteolytic mixture from the AFM chip surface, activated with the SuccBB crosslinker, after the immobilization of BSA from its 10^−7^ M solution (x = 3); a proteolytic mixture from the AFM chip surface modified with the SuccBB crosslinker, after the immobilization of BSA from its 10^−9^ M solution (x = 4). For y, a value of 1 or 2 was assigned depending on the presence or absence of the alkylation and reduction step in the sample preparation: y = 1 if there was the alkylation and reduction step; y = 2 if there was no alkylation and reduction step.

## 5. Conclusions

Herein, the optimal conditions for the MS identification of BSA immobilized on mica surfaces modified with two types of crosslinkers, succinimide and benzophenone crosslinkers (DSP and SuccBB, respectively), have been found. In our previous paper, we focused on the AFM detection of the protein captured on the chip surface at low (nanomolar) concentrations [8]. Therein, we reported that the use of DSP for capturing albumin from its 10^−9^ M solution in water onto the surface of an AFM chip was unsuccessful, thereby not allowing us to perform its MS identification [8]. In the present research, we have used different experimental conditions. Under these conditions, at a 10^−7^ M concentration of albumin in the PBSD buffer, both crosslinkers have been found suitable for the covalent capturing of BSA on the AFM chip surface. In this way, an important methodological issue of albumin capture onto the atomically smooth surface of DSP-activated AFM chips has been solved. This has allowed us to focus on the MS identification of the captured protein and to investigate how the crosslinker type influences the MS identification of the chip-captured protein. Namely, SuccBB was found to be more efficient with respect to its MS identification; upon the use of SuccBB, the protein captured onto the AFM chip surface was visualized in the form of layers, and more peptides were identified in the proteolytic mixture on the surface in MS analysis. In the case of DSP, a lower efficiency of protein capturing was observed. This can be explained by electrostatic repulsion effects, which prevented BSA molecules from approaching the substrate surface [8], and the specificity of the crosslinker for primary amine groups on the protein globule’s surface. It should be emphasized that, despite a lower capturing efficiency, the use of DSP does not require additional equipment for the UV irradiation of the AFM chip throughout protein capturing, providing a simpler realization of the experiment.

The results reported herein can be applied in state-of-the-art systems for the inventory of proteins. The composition of peptides in MS analysis, depending on the type of crosslinker used, should be taken into consideration in highly sensitive, targeted proteomic analysis upon selection of proteotypic peptides. Moreover, the data on how the crosslinker type influences the efficiency of protein capturing can be of interest for researchers developing new protein immobilization strategies for application in biosensor systems. Furthermore, these data should be taken into consideration in the development of highly sensitive diagnostic systems employing chip-immobilized proteins.

## Figures and Tables

**Table 1:** Results of MS analysis: number of measured m/z corresponding to BSA peptides.

	Sample Series/Protein Concentration	Alkylation	Number of Samples per Series	Average Number of Measured Masses of Peptides from the Peptide List *	Peptide List
1	10^−7^ M BSA solution (no AFM chip immersed)	Yes	3	12 ± 3	Peptoset 1_1
		No	3	11 ± 1	Peptoset 1_2
2	AFM chip, DSP crosslinker/10^−7^ M BSA	Yes	3	3 ± 1	Peptoset 2_1
		No	3	5 ± 3	Peptoset 2_2
3	SucBB crosslinker/10^−7^ M BSA	Yes	3	9 ± 8	Peptoset 3_1
		No	3	10 ± 6	Peptoset 3_2
4	SucBB crosslinker/10^−9^ M BSA	No	3	3 ± 2	Peptoset 4_2
5	Control 1 (blank)	No	3	0	Peptoset 5_2
6	Control 2	No	3	3 ± 1	Peptoset 6_2
7	Control 3	No	3	3 ± 1	Peptoset 7_2

* The average number is calculated as the arithmetic mean. The standard deviation is calculated as the degree of deviation of data from the mean (square root of the variance).

## Data Availability

Correspondence and requests for materials should be addressed to A.I.G.

## Supplementary Materials

The following supporting information can be downloaded at: https://www.mdpi.com/article/10.3390/ijms24108999/s1.
