# Supplementary material for: Mass Spectrometric Identification of BSA Covalently Captured onto a Chip for Atomic Force Microscopy
Source: Int J Mol Sci. 2023 May 19;24(10):8999. doi: 10.3390/ijms24108999 (PMC10219506; doi:10.3390/ijms24108999)
Supplement: Supplementary file 1 [file ijms-24-08999-s001.zip › ijms-2366330-supplementary.pdf]

Supplementary Material to

Mass Spectrometric Identification of BSA Covalently Captured onto a Chip for Atomic Force Microscopy

by

Arina I. Gordeeva, Anastasia A. Valueva, Maria O. Ershova, Elizaveta E. Rybakova, Ivan D. Shumov, Andrey F. Kozlov, Vadim S. Ziborov, Maria G. Zavialova, Victor G. Zgoda, Yuri D. Ivanov, Alexander I. Archakov and Tatyana O. Pleshakova*

Institute of Biomedical Chemistry (IBMC), 119121, Moscow, Russia
*Correspondence: topleshakova@yandex.ru; Tel.: +7 (499) 246-3761

In order to analyze the data obtained, the composition of peptides in each peptoset, obtained using either DSP (peptoset 2_2) or SuccBB (peptoset 3_2) crosslinker, or 10^{-7} M BSA solution is listed in Table S1.

Table S1. Results of MALDI–MS analysis obtained for peptoset 3_2, peptoset 2_2, and 10^{-7} M protein solution: m/z values of the identified peptides.

**Peptoset 3_2**	**Peptoset 2_2**	**10^{-7} M BSA Solution**
665.3287	689.3729	665.3287
689.3729	927.49344	689.3729
712.3736	1163.6306	927.4934
818.4254	1479.7954	1163.6306
922.488	1567.7427	1249.6211
927.49344	1955.9379	1283.7106
1050.4924		1439.8117
1249.6211		1479.7954
1305.7161		1567.7427
1479.7954		1639.9377
1511.8427		1955.9379
1567.7427		2045.0279
1639.9377		
1888.9268		

In order to additionally confirm the identification of the protein, MS/MS analysis of BSA peptides was performed. Figure S1 displays MS/MS spectra obtained upon analysis of 10^{-7} M BSA solution. Mass spectra of two BSA peptide precursor ions YLYEIAR (Figure S1a) and KVPQVSTPTLVEVSR (Figure S1b) are shown. The same peptides have been identified in the case of analysis of peptide eluates from SuccBB-activated AFM chips (see Table S1 above).

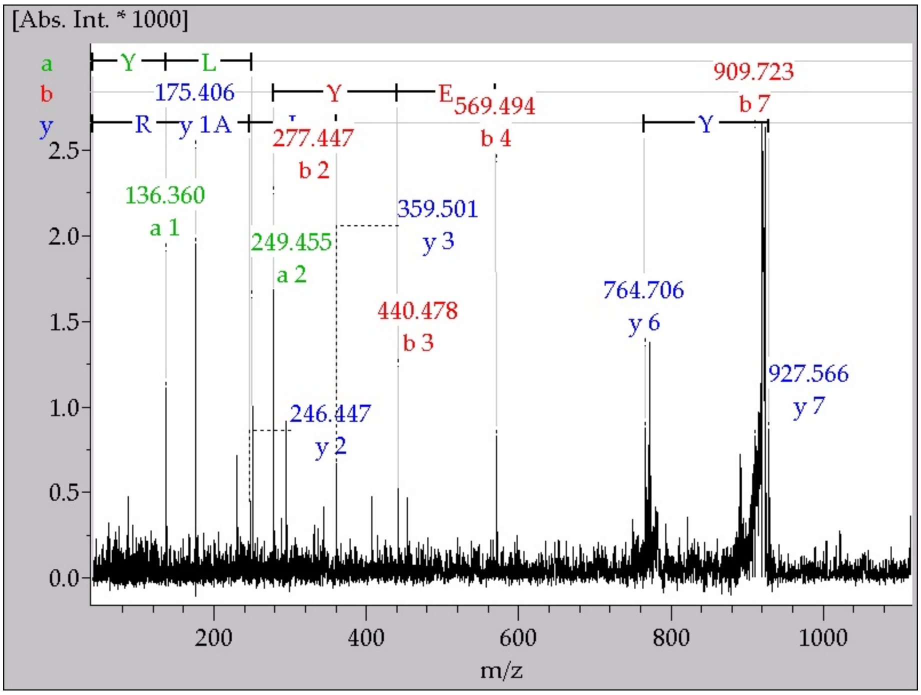

(a)

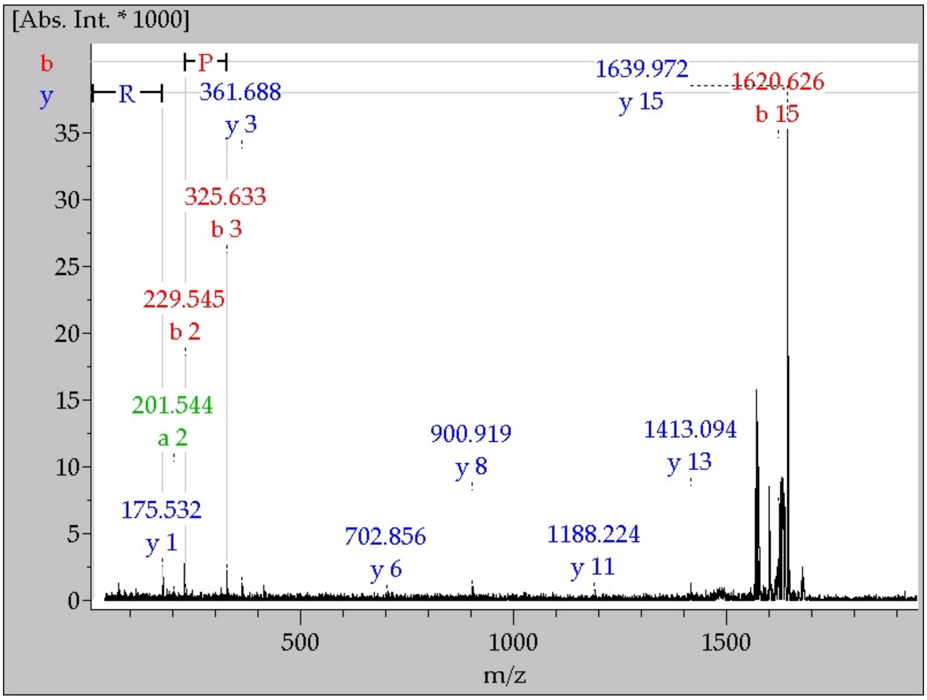

(b)

Figure S1. MALDI-TOF/TOF MS/MS spectrum of BSA molecular ions with amino acid sequence YLYEIAR and m/z 927.49344 (**a**) and with amino acid sequence KVPQVSTPTLVEVSR and m/z 1639.9377 (**b**). Coloured symbols indicate peaks, corresponding to Y-, a-, and b-ions, which result from the fragmentation of this structure (marked in blue, green, and red, respectively).
